# Transcriptomic response of *Pinus massoniana* to infection stress from the pine wood nematode *Bursaphelenchus xylophilus*

**DOI:** 10.1007/s44154-023-00131-z

**Published:** 2023-11-22

**Authors:** Yibo An, Yongxia Li, Ling Ma, Dongzhen Li, Wei Zhang, Yuqian Feng, Zhenkai Liu, Xuan Wang, Xiaojian Wen, Xingyao Zhang

**Affiliations:** 1 Key Laboratory of Forest Protection of National Forestry and Grassland Administration, Ecology and Nature Conservation Institute, Chinese Academy of Forestry, l00091, Beijing, China; 2 Co-Innovation Center for Sustainable Forestry in Southern China, Nanjing Forestry University, Nanjing, 210037 China; 3 Chongqing Forestry Investment and Development Co., Ltd., National Forestry and Grassland National Reserve Forest Engineering Technology Research Center, Chongqing, 401120 China; 4 Northeast Forestry University, College of Forestry, Harbin, 150040 China

**Keywords:** *Pinus massoniana*, *Bursaphelenchus xylophilus*, Transcriptome, WGCNA

## Abstract

The pinewood nematode (PWN) *Bursaphelenchus xylophilus* is a forestry quarantine pest and causes an extremely dangerous forest disease that is spreading worldwide. Due to the complex pathogenic factors of pine wood nematode disease, the pathogenesis is still unknown. *B. xylophilus* ultimately invades a host and causes death. However, little is known about the defence-regulating process of host pine after infection by *B. xylophilus* at the molecular level. Therefore, we wanted to understand how *Pinus massoniana* regulates its response to invasion by *B. xylophilus*. *P. massoniana* were artificially inoculated with *B. xylophilus* solution, while those without *B. xylophilus* solution were used as controls. *P. massoniana* inoculated with *B. xylophilus* solution for 0 h, 6 h, 24 h, and 120 h was subjected to high-throughput sequencing to obtain transcriptome data. At various time points (0 h, 6 h, 24 h, 120 h), gene transcription was measured in *P. massoniana* inoculated with PWN. At different time points, *P. massoniana* gene transcription differed significantly, with a response to early invasion by PWN. According to Gene Ontology (GO) classification and Kyoto Encyclopedia of Genes and Genomes (KEGG) enrichment analysis, *P. massoniana* response to PWN invasion involves a wide range of genes, including plant hormone signal transformation, flavonoid biosynthesis, amino sugar and nucleoside sugar metabolism, and MAPK signalling pathways. Among them, inoculation for 120 hours had the greatest impact on differential genes. Subsequently, weighted gene coexpression network analysis (WGCNA) was used to analyse transcriptional regulation of *P. massoniana* after PWN infection. The results showed that the core gene module of *P. massoniana* responding to PWN was “MEmagenta”, enriched in oxidative phosphorylation, amino sugar and nucleotide sugar metabolism, and the MAPK signalling pathway. MYB family transcription factors with the highest number of changes between infected and healthy pine trees accounted for 20.4% of the total differentially expressed transcription factors. To conclude, this study contributes to our understanding of the molecular mechanism of initial PWN infection of *P. massoniana*. Moreover, it provides some important background information on PWN pathogenic mechanisms.

**Supplementary Information:**

The online version contains supplementary material available at 10.1007/s44154-023-00131-z.

## Introduction

Pine wilt disease (PWD) is one of the greatest threats to pine trees and is spreading worldwide. In China, the PWD pathogen *Bursaphelenchus xylophilus* is listed as the only national first-class forest pest. Pinus provides us with many resources with ornamental, industrial and medicinal value. For pine trees, PWD is a devastating disease that is difficult to control. At present, there is no effective control method. Because most pine species in North America are resistant to pinewood nematode (PWN) *B. xylophilus*, infection due to geographical environment and other factors, PWN has little impact on pine trees (Jones et al. 2008). Nevertheless, in other PWN-infested locations, such as East Asia and Western Europe, pine trees exhibit great susceptibility to PWN, which has severely harmed forest ecology and resulted in significant financial losses for forest product businesses (Abelleira et al. 2011; Vicente et al. 2012; Inacio et al. 2015). In various locales, PWN has varying levels of virulence. In Japan, China, and the Iberian Peninsula, distinct PWN pathogenicity types have been discovered (Menendez-Gutierrez et al. 2021; Palomares-Rius et al. 2015; Ding et al. 2016). PWNs collected from the same geographic location also exhibit varying virulence levels within the same species or across different species (Akiba et al. 2012).

Additionally, these regions show an increase in the genetic diversity of these isolates over a long period of time (Valadas et al. 2013). In several nations, particularly in Japan and China, PWN has led to significant economic losses and harmed the environment of pine forests. In southern China, *Pinus massoniana* is a significant conifer that produces oleoresin and lumber. The most commonly affected tree species in China by pine wilt is *P. massoniana*. The State Forestry Administration of China stated in 2022 that from the disease’s initial detection in Nanjing, China, in 1982, it had spread to 19 administrative provinces and resulted in significant economic and ecological damage (Zheng et al. 2021; Li et al. 2021). Additionally, every year, there are more injured *P. massoniana* trees (Zhao et al. 2007). The major ascaroside pheromone component asc-C5 influences reproductive plasticity among isolates of invasive PWN in the process of invasion (Zhao et al. 2021). This result suggests that the plasticity of reproductive responses to PWN pheromones may increase its fitness in novel environments following introduction. The pine sawyer beetle *Monochamus alternatus* Hope (Coleoptera, Cerambycidae) is a key insect vector of the destructive forest pest PWN, which has caused widespread and destructive PWD throughout Asia and Europe (Kim et al. 2020). Recent studies have demonstrated that some insect-associated bacteria might decrease fungal toxicity and further undermine its biological control efficacy against *M. alternatus*. For example, associated bacteria (*Pseudomonas* and *Serratia*) of a pine sawyer beetle confer resistance to entomopathogenic *Beauveria bassiana* via fungal growth inhibition (Deng et al. 2022).

The MYB (myoblast) transcription factor family is a class of transcription factors containing the MYB domain and is widely present in plants. When plants are subjected to external biological or abiotic stress, MYB family members recognize and bind to specific gene promoter motifs to activate transcription, improving plant stress resistance. For example, the MYB promoter conserved binding motif (A/C) ACC (A/T) A (A/C) C, AtMyb1 motif (A/C) TCC (A/T) ACC, AtMyb2 motif TAAC (G/C) GTT, AtMyb3 motif TAACTAAC, AtMyb4 motif A (A/C) C (A/T) A (A/C) C (Sablowski et al. 1994; Menkens and Cashmore 1994; Prouse and Campbell 2012; Wang et al. 2020), and many R2R3-MYB transcription factors bind to DNA sequences that are rich in adenine A and cytosine C (Gomez-Maldonado et al. 2004) and do not or rarely contain guanine G (Prouse and Campbell 2013). MYB activates transcription by binding to plant stress response genes, improving tolerance and attenuating stress damage. In apple, MYB10 mainly regulates accumulation of antioxidant anthocyanins by binding to the R1 motif (ACTGGTAGCTATT) to activate its transcription. In other gene-mutated apple strains, a specific motif R6 (ACTGGTAG (T) C (T) TAT (A) T) was found in the MYB10 promoter, resulting in stronger activation signals and more anthocyanin accumulation (Brendolise et al. 2017). In apple under low-temperature stress, MdMYB88/MdMYB124 activates transcription by binding to specific sequences of low-temperature responsive genes, such as the motif (AACCG) of the MdCCA1 promoter and the cis element (AACCG) of the MdCSP3 promoter, thereby improving low-temperature tolerance (Xie et al. 2018).

The pathogenesis of PWD has been extensively studied. However, the mode of transmission is diverse, and human factors are uncontrollable. In addition, resumption of production will inevitably lead to a large number of trade activities, with a great impact on transmission of PWD. The molecular basis of the interaction between PWN and its host pine is not fully understood (Kikuchi et al. 2011). In China, some pine varieties have strong resistance to PWN. Therefore, researchers also study the molecular mechanism of the response to PWN between resistant and susceptible species (Gaspar et al. 2020; Gaspar et al. 2017; Santos et al. 2012). However, relevant studies on inoculation of PWN in *P. massoniana* only involve relatively late interaction times, such as 15 dpi and 30 dpi. To better understand the transcriptional response of *P. massoniana* after being invaded by PWN and considering that the main activity site for PWN reproduction and diffusion is the host’s stem, this study explored the transcriptional status of the stem tissue of *P. massoniana* inoculated with PWN at 6 h, 24 h, and 120 h.

High-throughput sequencing is a powerful tool to identify differential transcription of the whole genome under different conditions. Considering the importance of *P. massoniana*, we adopted the method of artificial inoculation to analyse the gene expression changes in *P. massoniana* infected by PWN at three time points. The purpose of this study was to promote understanding of the molecular response of *P. massoniana* to early infection by PWN. Weighted gene coexpression network analysis (WGCNA) was used to study the complex relationship between genes and phenotypes, the core genes at different stages were screened by constructing protein–protein interaction network analysis, and their role in pathogenesis was determined. The findings will be helpful for research on the early diagnosis of PWN and provide important reference data for research on the pathogenesis of PWN.

## Results

### RNA-sequencing analysis

**Fig. 1:**
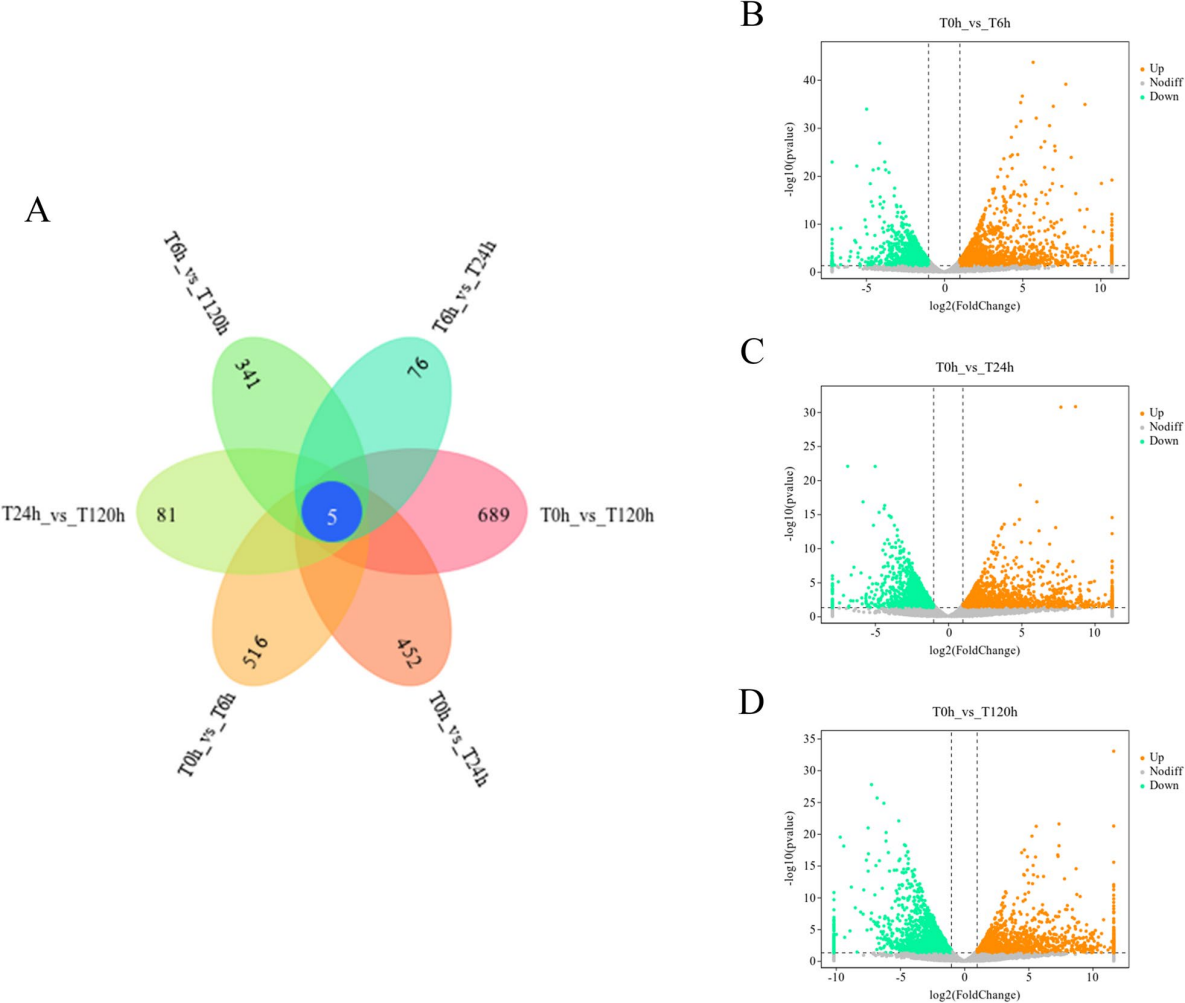
Transcriptomic analysis of differential gene distribution. **A**, Venn diagram of different genes in samples at different time points. **B**-**D**, Volcano map of different genes in samples at different time points

Differential expression of genes was analysed by DESeq to determine how transcriptome data changed during the early invasion of pine wood nematodes into host pine trees. During the screening procedure, the following conditions were needed: expression difference multiple log2-fold change > 1 and significant *p* < 0.05. Genes with differential expression were identified in the four samples T0h, T6h, T24h, and T120h, and they were compared in pairs. The number of differentially expressed genes between the groups is shown in the Wayne plot in Fig. 1A, with the highest number of differentially expressed genes between the two groups being T0h_VS_T120h; 689 differential genes were identified. The minimum number of differentially expressed genes was T6h_VS_T24h, and 76 differential genes were identified. A total of 5 unigenes showed conservative expression in the samples at the four time points (Fig. 1A), namely, PITA_39865, PITA_39318, PITA_09037, PITA_37081 and PITA_50,920. Among them, PITA_39865 belongs to the cysteine-rich protein family regulated by GASA gibberellin; PITA_39318 belongs to the PC-Esterase protein family; and PITA_37081 belongs to the L-ascorbate oxidase (EC 1.10.3.3) protein family. PITA_ 09037 and PITA_ 50,920 have an unknown functional domain.

### Analyses of DEG functional enrichment

**Fig. 2:**
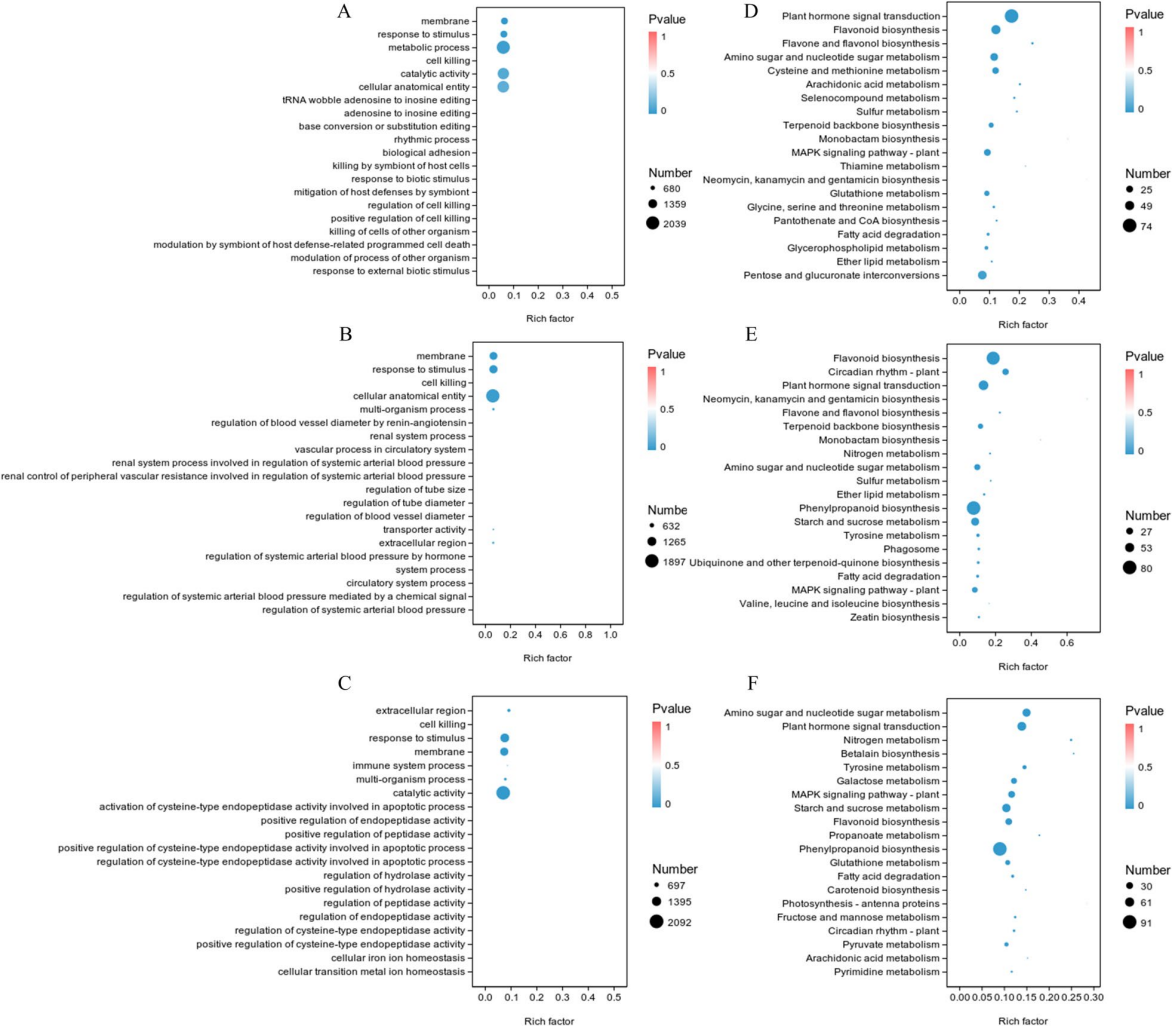
Map of GO classification enrichment and KEGG enrichment factors of differentially expressed genes. **A-C**, Comparison of two different genes at different time points: (A) T0h_VS_ T6h; (B) T0h_VS_ T24h; and (C) T0h_ VS_ T120h. **D**-**F**, KEGG classification enrichment comparison of two different genes at different time points: (D) T0h_VS_ T6h; (E) T0h_VS_ T24h; and (F) T0h_VS_ T120h

Our study covers three aspects of molecular function, cellular component, and biological process using GO enrichment analysis for differentially expressed genes (DEGs) in *P. massoniana*-pine wood nematode interactions. After 6 hours of interaction, we found significant enrichment (*p* < 0.05) in several categories, including 622 upregulated genes and 444 downregulated genes in cellular component (CC), 686 upregulated genes and 389 downregulated genes in response to stimulation (BP), 1238 upregulated genes and 801 downregulated genes in metabolic process (BP), and 10 upregulated genes in cell killing (BP) (Fig. 2 A, Supplementary Table 1, Sheet 1). When the interaction between *P. massoniana* and pine wood nematodes lasted for 24 hours, there were significantly enriched (*p* < 0.05) members (CC), including 573 upregulated genes and 541 downregulated genes; response to stimulation (BP), with 680 upregulated genes and 480 downregulated genes; cell killing (BP), with 13 upregulated genes and 0 downregulated genes; cellular anatomical entity (CC), with 982 upregulated genes and 915 downregulated genes; and multiorganizational process (BP), with 230 upregulated genes and 116 downregulated genes (Fig. 2 B, Supplementary Table 1, Sheet 2). When *P. massoniana* interacted with pine wood nematodes for 120 hours, the enrichment degree was extremely significant (*p* < 0.01) in the extracellular region (CC), with 245 upregulated genes and 240 downregulated genes; cell killing (BP), with 23 upregulated genes and 1 downregulated gene; response to stimulation (BP), with 682 upregulated genes and 654 downregulated genes; membership (CC), with 562 upregulated genes and 677 downregulated genes; immune system process (BP), with 111 upregulated genes and 49 downregulated genes; multiorganizational process (BP), with 229 upregulated genes and 183 downregulated genes; and cationic activity (MF), with 974 upregulated genes and 1118 downregulated genes (Fig. 2 C, Supplementary Table 1, Sheet 3). GO analysis showed that compared to the T0h sample, enrichment of differentially expressed genes in the T120h sample was more significant than at other time points, and the number of differentially expressed genes was also highest in the molecular function category.

We analysed KEGG pathway enrichment of DEGs over various time periods, choosing the top 20 pathways to explore the role that the DEGs play in the early pine wood nematode invasion of *P. massoniana*. By comparison with the T0h sample, the top 5 pathways with the highest abundance were found to be “plant hormone signal transduction”, “flavonoid biosynthesis”, “flavonoid and flavonol biosynthesis”, “amino sugar and nucleoside sugar metabolism”, and “cysteine and methionine metabolism” when pine wood nematodes invaded the host for 6 hours (Fig. 2D, Supplementary Table 2, Sheet 1). When pinewood nematodes invaded the host for 24 hours, the most abundant top 5 pathways were “flavonoid biosynthesis”, “circadian rhythm plant”, “plant hormone signal transformation”, “neomycin, kanamycin and gentamicin biosynthesis”, “flavone and flavonol biosynthesis” (Fig. 2E, Supplementary Table 2, Sheet 2). When pine wood nematodes invaded the host for 120 hours, the most abundant top 5 pathways were “amino sugar and nucleate sugar metabolism”, “plant hormone signal transformation”, “nitrogen metabolism”, “betalain biosynthesis”, and “tyrosine metabolism” (Fig. 2F, Supplementary Table 2, Sheet 3). Accordingly, the KEGG results suggest that when the PWN invades early in a plant, the host mainly responds through plant hormone signal transduction pathways, flavonoid metabolism pathways, and amino sugar and nucleotide sugar metabolism pathways, which provide the host with defences and immunity against PWN.

### Key genes and modules of the *P. massoniana* response to pine wood nematode infection defined by WGCNA

By using weighted gene coexpression network analysis (WGCNA), gene interaction networks can be constructed to identify gene modules and their core genes. By analysing RNA-seq data, we identified key genes and modules involved in the response of *P. massoniana* to pine wilt infection. First, cluster analysis was performed on 12 samples, and there were no outliers (Supplementary Fig. 1A). The soft threshold was calculated simultaneously, and 16 was used as the soft threshold for constructing the network (Supplementary Fig. 1B). Based on the soft threshold test, the enrichment trend of genes was normal (from left to right), with an R2 value of 0.78 and a slope value of − 1.33. Based on the results of the test, we proceeded to the next analysis (Supplementary Fig. 1C). A gene module consists of at least 30 genes, and each colour represents a gene module. Supplementary Fig. 1D shows that genes with a correlation coefficient greater than 0.75 are considered to be part of the same module. The module trait correlation analysis was conducted on 12 samples, and four modules reached a significant level (turquoise, red, grey, and magenta), which correlated significantly with the 0 h, 6 h, 24 h, and 120 h samples. These modules contain 69 genes (magenta) to 2516 genes (turquoise) (Supplementary Table 3). Among them, 120 h MEmagenta is the most relevant gene module. In terms of gene-to-module correlation (MM) and gene-to-phenotype correlation (GS), it was verified that the magenta module in the 120 h sample was consistent with the core modules.

**Fig. 3:**
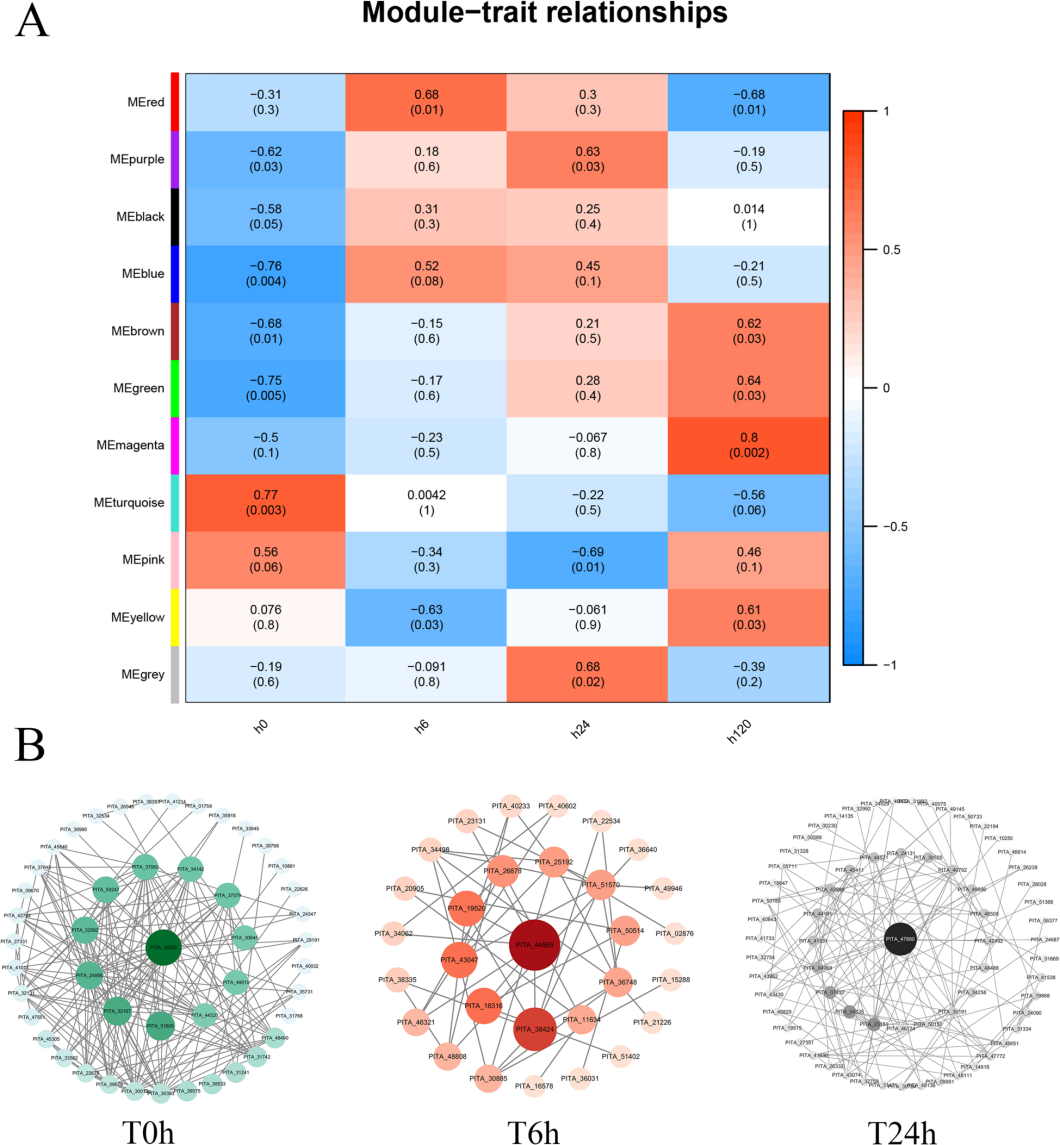
Correlation between gene modules and phenotypes and construction of a protein–protein interaction network at different time stages. **A**, Module-phenotypic correlation network of differential genes (0 h, 6 h, 24 h, 120 h) in 4 sample groups. **B**, Gene protein interaction network analysis of core modules at different time points. The dot in the middle represents the core gene. ME: module eigengene

Based on module-trait correlation analysis for 12 samples, four modules reached significant levels (turquoise, red, grey, and magenta) and correlated significantly with the 0 h, 6 h, 24 h, and 120 h samples (Fig. 3 A). Regarding gene modules, the MEmagenta module at 120 hours was most relevant. Despite the early invasion of pine nematodes (5 days ago), the transcriptional profile of *P. massoniana* was unclear. *Pinus sylvestris* core gene screening is therefore critical for the early diagnosis of pine nematode disease and the development of control strategies, and we constructed a protein interaction network (PPI) to further mine the core module genes at each time point (Fig. 3 B). Gene networks were constructed by using the STRING database and mediocentrality (betweenness centrality) scores of the genes in modules. In the MEturquoise module, the top 47 core genes were identified; in the MEred module, the top 30 core genes were identified; and in the MEgrey module, the top 68 core genes were identified. Although the most significant correlation between genes in the MEmagenta module and treatment time was found in the 120 h sample at all time points (Fig. 3 A), the gene interactions in the MEmagenta module did not appear in the STRING database, so only gene interactions in the 0 h, 6 h, and 24 h samples are displayed here. *PITA_ 38,325* is the core gene of the T0h sample; *PITA_ 44,565* is the core gene of the T6h sample; and *PITA_ 47,880* is the core gene of the T24h sample.

### Gene enrichment analysis in the core module

**Fig. 4:**
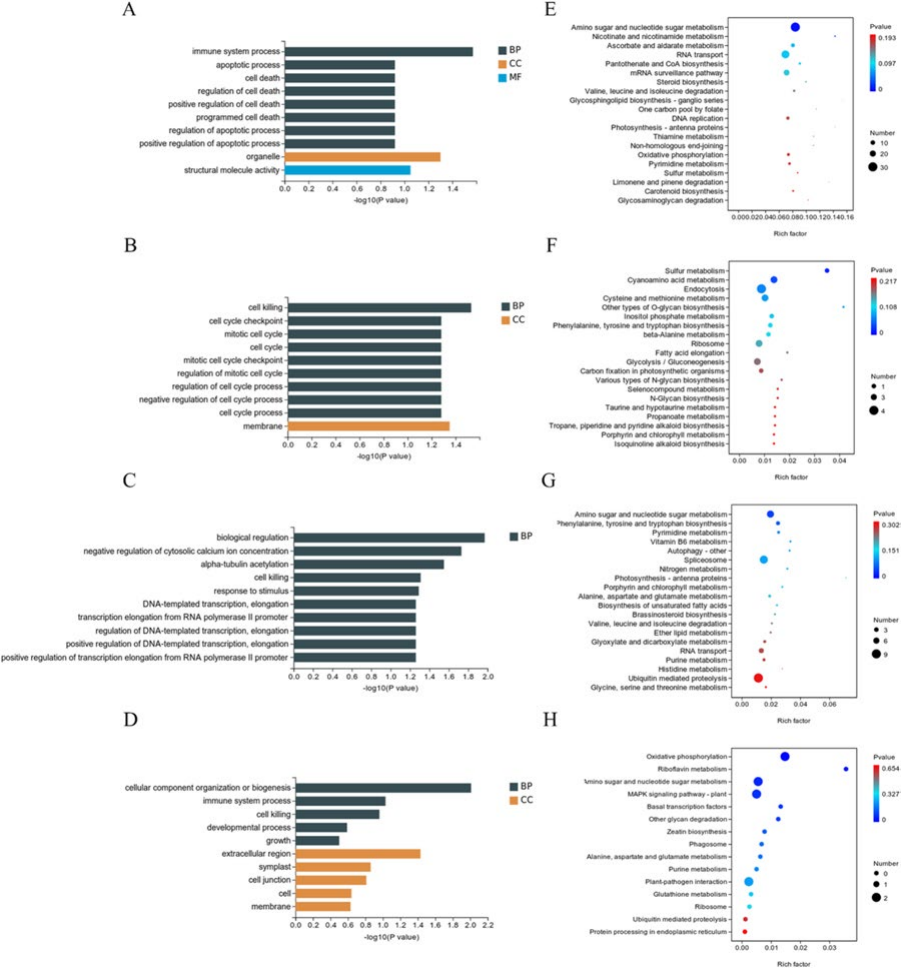
Gene enrichment analysis of core modules at different time points. **A**-**D**, GO classification and enrichment analysis of the MEturquoise, MEred, MEgrey and MEmagenta modules. **E**-**H**, KEGG enrichment analysis of the MEturquoise, MEred, MEgrey and MEmagenta modules

GO enrichment and KEGG enrichment methods were used to annotate and enrich the core genes in these four modules. The genes of the MEturquoise module were mainly enriched in flavonoid biosynthesis, stilbenes, diarylheptanes, gingerol biosynthesis, and zeatin biosynthesis (Fig. 4A, E). The genes of the MEred module were mainly enriched in the sulfur metabolism system, nitrogen metabolism, and folate synthesis (Fig. 4B, F). The genes of the MEgrey module were mainly enriched in monoterpene biosynthesis, fatty acid elongation, and vitamin B6 metabolism (Fig. 4C, G). The genes of the MEmagenta module were mainly enriched in oxidative phosphorylation, amino sugar and nucleotide sugar metabolism, and the plant MAPK signalling pathway (Fig. 4D, H). Therefore, we hypothesize that the core pathways of early Sargassum pine in response to pine nematodes may be related to the synthetic transport processes of sugar metabolism and the activation of antiretroviral factors. In response to pine wood nematode invasion, the core genes of the *P. massoniana* response play a linkage and defence function.

### RNA-seq validation by qRT–PCR

**Fig. 5:**
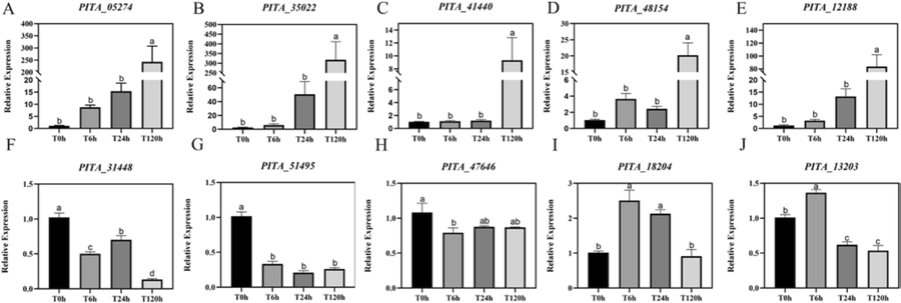
qRT–PCR detection of core differential genes in the MEmagenta module. Different lowercase letters on the column indicate significant differences (*p* < 0.05)

By using WGCNA, the MEmagenta module of the 120 h sample was found to have the strongest relationship between nematode treatment time and *P. massoniana* transcription (Fig. 3A). Heatmap analysis was performed on the genes extracted from the MEmagenta module (Supplementary Fig. 2). Based on the MEmagenta module (Supplementary Fig. 3), 10 candidate genes with differential expression were randomly selected and analysed using qRT–PCR. Based on our transcriptome sequencing results (Fig. 5), the gene expression changes are consistent with our RNA-seq data.

### Analysis of transcription factors

**Fig. 6:**
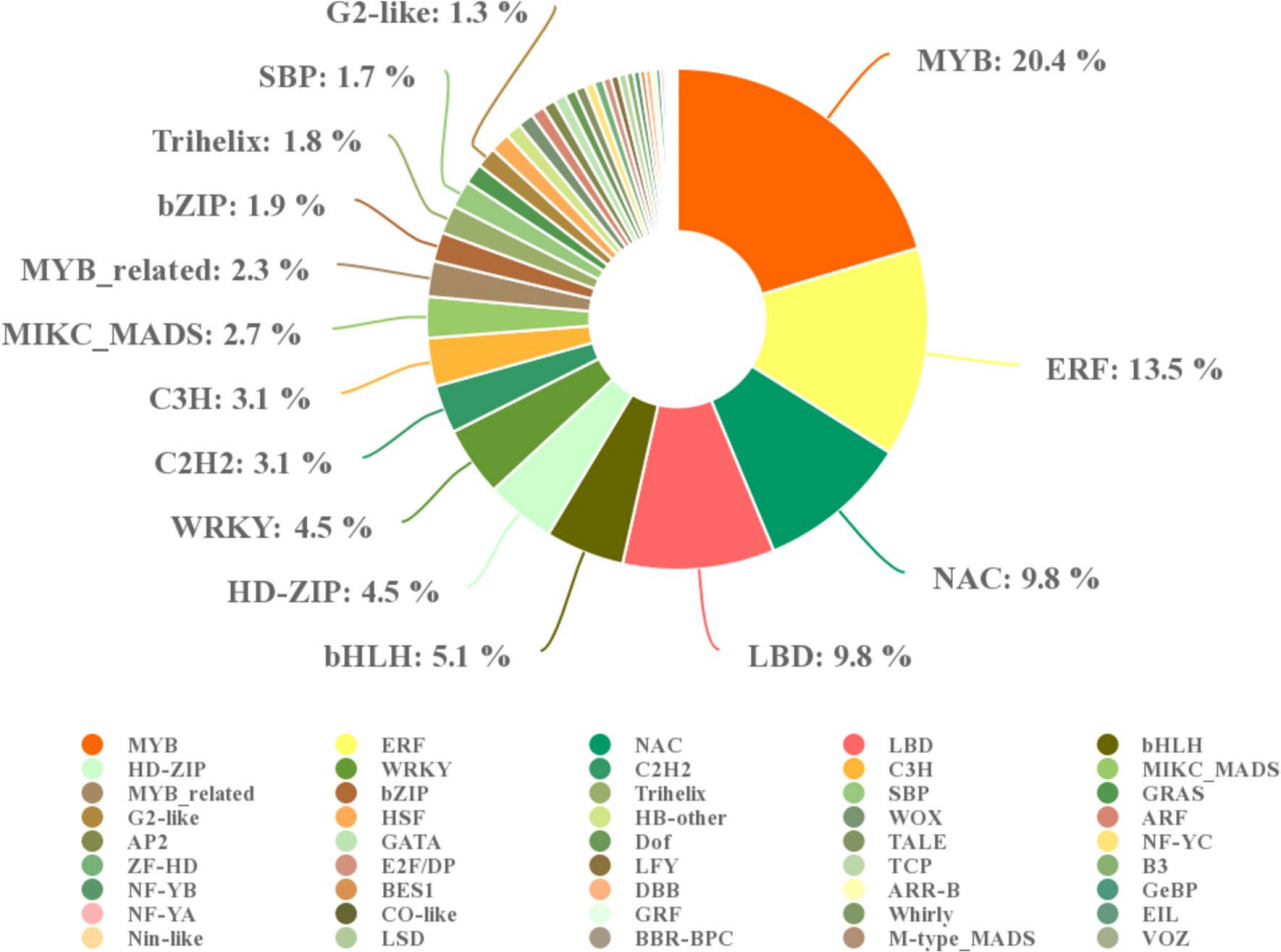
Quantitative statistics of differentially expressed transcription factors in the transcriptome

On the basis of the differentially expressed transcription factors in the transcriptome data, the transcription factor families involved in the primary response of *P. massoniana* include MYB, ERF, NAC, LBD, and bHLH, comprising 20.4%, 13.5%, 9.8%, 9.8% and 5.1%, respectively, of all transcription factors that were differentially expressed. There is a strong correlation between each of these transcription factors and host stress resistance (Fig. 6).

## Discussion

Research has been conducted in recent years to explore the pathogenesis of pine wood nematodes using multiomics methods in addition to traditional pathological methods, such as proteomics (Shinya et al. 2013), metabonomics (An et al. 2022) and transcriptomics (Chen et al. 2021a). As a result of the application of omics methods, molecular regulatory mechanisms in hosts and pathogenic nematodes can be comprehensively and rapidly examined. By using transcriptomes, researchers have primarily focused on the nematode itself (Lu et al. 2020; Chen et al. 2021b; Guo et al. 2020; Li et al. 2019). There is also a portion of research that focuses on transcriptional changes in host pine trees(Gaspar et al. 2020; Liu et al. 2017). Gene Ontology (GO) enrichment analysis of DEGs revealed that the most significant biological processes were “syncytium formation” in the resistant phenotype and “Response to stress” and “Terpenoid biosynthesis” in the susceptible phenotype at 1 and 15 dpi, respectively. The results of this study show that the most abundant TOP5 pathway is “flavonoid biosynthesis”, “circadian rhythm plant”, “plant hormone signal transformation”, “neomycin, kanamycin and gentamicin biosynthesis”, and “flavone and flavonol biosynthesis” at 1 dpi (Fig. 2E, Supplementary Table 2, Sheet 2).

Transcriptomes can help us obtain DEGs to reflect host gene responses in pathogen interactions (Hirao et al. 2012). We found that there has been limited research on the molecular mechanisms involved in *P. massoniana*’s response to PWN infection; especially in the first 120 hours after inoculation of pine wood nematodes with *P. massoniana*, there is no relevant data published. As a result, there is a lack of research on the response mechanism of early Masson pine to PWN. RNA-Seq analysis was used to characterize the transcriptome profile of *P. massoniana* after inoculation with pinewood nematodes.

The susceptibility of plant species to insects and pathogens is influenced by qualitative and quantitative differences in expression of activated genes (Thompson and Goggin 2006). According to GO classification of DEGs, there is a significant difference in activated defence genes between resistant or susceptible tree species when resistant or susceptible tree species are infected by pine wood nematodes. There was a significant difference in differentially expressed genes between the T120h and T0h samples, and the number of differentially expressed genes was also highest among the molecular functional categories compared to all other time points. The cell membrane component “membrane” had the highest degree of differential DEG enrichment (Fig. 2A). It is essential for pine wood nematodes to insert their mouth needles into parenchymal cells to obtain the nutrition necessary for reproduction. Furthermore, it is hypothesized that the nematode secretes effector proteins or small molecules during this process. Several studies have indicated that some signals are transmitted from nematode secretions to host cells, resulting in enrichment of genes classified as “membrane” genes. Previous studies have found that effector proteins secreted by pinewood nematodes during the pathogenic process can also damage the host’s immune system (Kikuchi et al. 2009) and stimulate its immune response (Hu et al. 2019; Zhao et al. 2020). Based on the enrichment of different genes in different stages of inoculation through KEGG pathway analysis, *P. massoniana* responds mainly to pine wood nematode invasion by signalling plants with plant hormones and by metabolizing terpenes via flavonoid biosynthesis and flavonoid metabolism (Fig. 2B). Researchers have discovered that PWNs secrete a large amount of the thaumatin-like protein BxTH1 (Shinya et al. 2013), which can induce cell death (Kirino et al. 2020). Additionally, a previous study found that interfering with pinewood nematode secretion protein genes delayed the onset of pine tree disease and reduced pine wood nematode toxicity (Meng et al. 2019). According to GO classification enrichment and KEGG enrichment of differential genes in *P. massoniana* infected with pine wood nematodes, early pine wood nematodes may exchange signals with *P. massoniana* through secretion of many effector proteins, and when external signals are received, *P. massoniana* may respond.

In this study, the core gene module “MEmagenta” of early *P. massoniana* responding to pine wood nematodes was identified through analysis of transcriptome data. The genes closely related to this module were highly enriched in oxidative phosphorylation, amino sugar and nucleotide sugar metabolism, and the plant MAPK signalling pathway (Fig. 4H), indicating that *P. massoniana* may express some molecular signals, specifically the response of stress resistance factors to resist pine wood nematode invasion. In the transcriptome data, differentially expressed transcription factor families were mostly associated with MYB, ERF, and NAC (Fig. 6). Among the functions of MYB transcription factors in plants are transduction of hormone signals, response to abiotic stress, and regulation of pathogenic fungi (Katiyar et al. 2012). Disease resistance signals and plant defence responses are regulated by plant antifungal activity. In terms of plant disease resistance signal transmission, the Arabidopsis AtMYB44 gene can directly control expression of the WRKY70 gene in salicylic acid SA-mediated and jasmonic acid JA-mediated defence responses (Shim et al. 2013). In Arabidopsis, MYB72 plays an important role in signal transduction during the early stages of systemic resistance induced by rhizosphere bacteria. During stripe rust stress, the MYB gene in wheat is upregulated, thereby regulating plant disease resistance. Consequently, MYB plays an important role in wheat resistance to stripe rust (Al-Attala et al. 2014). In this study, a transcription factor in the MYB family was found to have a significant impact on *P. massoniana*’s response to pine wood nematode invasion. It is speculated that *P. massoniana* activates MYB transcription factors to enhance its response to pine wood nematode invasion. To improve plant tolerance to stress damage, MYB activates transcription by binding to the sequence of plant stress response genes. In apple, MYB10 is primarily responsible for regulating accumulation of antioxidant anthocyanins through binding to the R1 motif (ACTGGTAGCTATT), activating its transcription. MYB10 binds to the specific motif R6 (ACTGGTAG (T) C (T) TAT (A) T) in other gene-mutated apple strains, leading to stronger activation signals and increased anthocyanin accumulation (Brendolise et al. 2017). By activating synthesis of phytoalexin 3-deoxyanthocyanidin at the site of primary infection, the MYB transcription factor of sorghum enhances the resistance of maize to verticillium wilt (Ibraheem et al. 2015). In wheat, the R2R3 MYB transcription factor TaPIMP1 regulates resistance to *Bipolaris sorokiniana*, which causes wheat root rot (Zhang et al. 2012). Since PWN invasion may activate differential expression of the stress resistance transcription factor family MYB in *P. massoniana*, why cannot resistance signal transduction be used to regulate downstream stress resistance genes to prevent invasion? According to some researchers, PWNs may escape the immune defence of *P. massoniana* by secreting effector proteins to ensure their reproduction and dissemination. Therefore, subsequent experiments need to focus on the communication between the host and the pine wood nematode during the interaction process, specifically the cellular level interaction response, as this may be the answer to the immune defence of the pine wood nematode escaping the host *P. massoniana*. During the pathogenic process of pine wood nematodes, it is crucial to analyse the interaction mechanism between *P. massoniana* and pinewood nematodes to determine the molecular mechanism of *P. massoniana*’s response.

## Materials and methods

### Sample processing

In the greenhouse, three-year masson pine potted seedlings were drilled at an angle of 45° below the main stem at 10–15 cm above the ground; 40 μL of pinewood nematode liquid (250/μL) was injected into the hole with a pipette, and the hole was quickly wrapped with a sealing film to ensure no evaporation. The negative control of the experiment was drilled seedlings that were not inoculated with pine wood nematodes. After inoculation with pine wood nematode solution for 0, 6, 24, and 120 hours, the stem tissues at 3 cm above and below the inoculation point were taken as experimental samples, with three biological replicates for each treatment. Approximately 1 g of cut slices was transferred to a 1.5 mL tube for storage. All samples were stored at − 80 °C and later sent to Shanghai Parsono Biotechnology Co., Ltd. for eukaryotic transcriptome sequencing.

### RNA extraction and detection

Total RNA quality detection: Concentration and purity were detected using a NanoDrop spectrophotometer (Thermo Scientific NanoDrop 2000). Integrity was assessed using RNA-specific agarose electrophoresis or a 2100 assay (Agilent 2100 Bioanalyzer. RNA 6000 Nano kit 5067–1511).

### Library construction and quality inspection

Total RNA with a total amount of ≥1 μg was selected, and the NEBNext Ultra II RNA Library Prep Kit for Illumina kit (strand-specific library building kit NEBNext Ultra Direct RNA Library Prep Kit for Illumina) was used. mRNA with polyA tails was enriched using oligo (dT) magnetic beads and then randomly disrupted using divalent cations. cDNA was synthesized using fragmented mRNA as a template and random oligonucleotides as primers. Purification of double-stranded cDNA followed by double-terminal repair and introduction of the ‘A’ base at the 3 ‘end and connection to sequencing connectors. AMPure XP beans were used to screen cDNA of approximately 400–500 bp, and PCR amplification was performed. The PCR product was purified again using AMPure XP beans, ultimately obtaining a library. An Agilent 2100 Bioanalyzer (Agilent, 2100) and Agilent High Sensitivity DNA Kit (Agilent, 5067–4626) were used for library quality testing. By using Pico Green to detect the total concentration of the library (Quantifluor-ST fluorometer, Promega, E6090; Quant-IT PicoGreen dsDNA Assay Kit, Invitrogen, P7589), quantitative detection of effective library concentration < Thermo Scientific StepOnePlus Real Time PCR Systems> was performed by qPCR. After homogenization of multiple DNA libraries and equal volume mixing, the mixed library was diluted and quantified before performing PE150 mode sequencing using an Illumina sequencer.

### Differential expression analysis

Differential expression analysis between two comparative combinations was performed using DESeq software (1.20.0). Differential analysis of gene expression was conducted using DESeq, and the screening conditions for differentially expressed genes were as follows: expression difference multiple | log2FoldChange | > 1, significance *p* < 0.05.

### Differential gene enrichment analysis

GO enrichment analysis was carried out with the top GO, and P was calculated by the hypergeometric distribution method (the standard of significant enrichment was *p* < 0.05) to determine the GO terms of significant enrichment for differential genes to determine their main biological functions. ClusterProfiler (3.4.4) software was used for KEGG pathway enrichment analysis, focusing on significantly enriched pathways with *p* < 0.05.

### Differential gene protein network interaction analysis

According to the STRING database (https://string-db.org/), protein interaction analysis was performed to reveal the interaction relationship between target genes. When the PPI information of the species is input, PPI pairs with differential genes and score > 0.95 are directly screened based on the results of gene differential expression analysis. When the network is too large or too small, the score value can be adjusted. When there is no PPI information for a species in the STRING database, similar species are selected to compare their protein sequences and then obtain interrelationships between their proteins. For transcriptome sequencing projects without a reference genome, similar species are selected to compare the unigene sequences of the species.

### WGCNA

Weighted Gene Coexpression Network Analysis (https://horvath.genetics.ucla.edu/html/CoexpressionNetwork) was used to explore the complex relationship between genes and phenotypes(Langfelder and Horvath 2008). When the independence exceeds 0.8, the appropriate power value is determined. For high reliability of the results, the minimum number of genes is set to 30. Meanwhile, genes with a correlation coefficient greater than 0.75 between modules are classified as the same module. The correlation between ME and traits was used to estimate module trait associations. The IC of each gene was calculated by adding the connection strength with other module genes and dividing this number by the maximum IC. The IC value is only defined for genes within a given module. IC measures the degree of connectivity or coexpression of a given gene relative to a specific module of genes. For each expression profile, GS is calculated as the absolute value of the Pearson correlation between the expression profile and each trait. MM is defined as the Pearson correlation between the expression profile and each ME.

### qRT–PCR detection of RNA-seq data

Randomly select genes from 10 core modules and calculate gene expression using 2^-∆∆Ct^(Schmittgen and Livak 2008), where ∆Ct = Ct target gene Ct internal reference gene, ∆∆ Ct = ∆Ct sample - ∆Ct average, and perform one-way ANOVA using SPSS 14.0 software(Heitkamp et al. 1990). GraphPad Prism 8.0.2 software (USA) was used for plotting to detect the reliability of the transcriptome data.

### Supplementary Information

**Additional file 1.**

**Additional file 2.**

**Additional file 3.**

**Additional file 4.**

## Abbreviations

PWN: Pinewood nematode
PWD: Pine wilt disease
DEGs: differential gene expression
CC: Cellular Component
BP: Biological Process
MF: Molecular Function
MM: Module membership
ME: module eigengene
GS: gene significance
PPI: Protein-Protein Interaction Networks
FC: Fold change
GO: Gene ontology
WGCNA: Weighted Gene Co-expression Network Analysis
KEGG: Kyoto Encyclopedia of Genes and Genomes
