# Supplementary material for: Transcriptomic response of Pinus massoniana to infection stress from the pine wood nematode Bursaphelenchus xylophilus
Source: Stress Biol. 2023 Nov 22;3(1):50. doi: 10.1007/s44154-023-00131-z (PMC10665292; doi:10.1007/s44154-023-00131-z)
Supplement: Supplementary file 3 — Additional file 3. [file 44154_2023_131_MOESM3_ESM.docx]

Additional file: **Supplementary Fig. 1** Data preprocessing for transcriptome analysis and WGCNA. **A**, The cluster analysis was conducted using 12 sample datasets. **B**, Calculation of the soft threshold for the model. **C**, Gene enrichment trend chart. **D**, Calculation of the correlation coefficient and cluster analysis. **E**, Analysis of gene-module correlations. **F**, Analysis of gene-phenotype correlations. **Supplementary Fig. 2** Heatmap of MEmagenta module core gene expression. **Supplementary Fig. 3** Ten genes from the MEmagenta module that exhibit differential expression at random were selected and analysed.


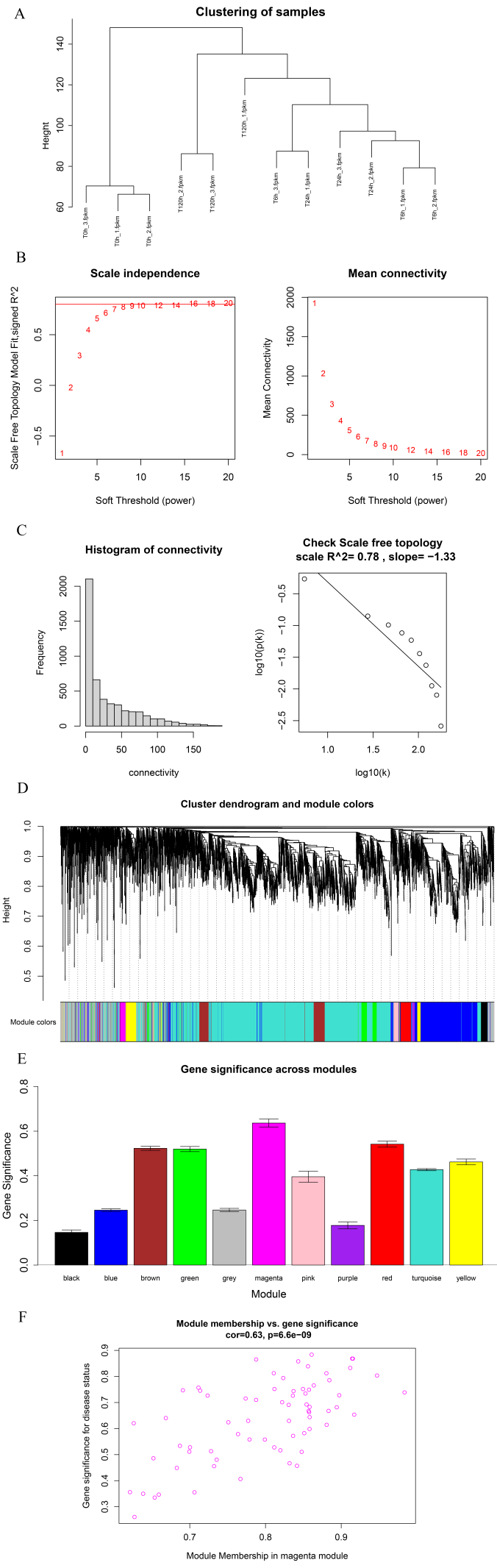


**Supplementary Fig. 1** Data preprocessing for transcriptome analysis and WGCNA. **A**, The cluster analysis was conducted on 12 sample datasets. **B**, Calculation of the soft threshold for the model. **C**, Gene enrichment trend chart. **D**, Calculation of the correlation coefficient and cluster analysis. **E**, Analysis of gene-module correlations. **F**, Analysis of gene-phenotype correlations.


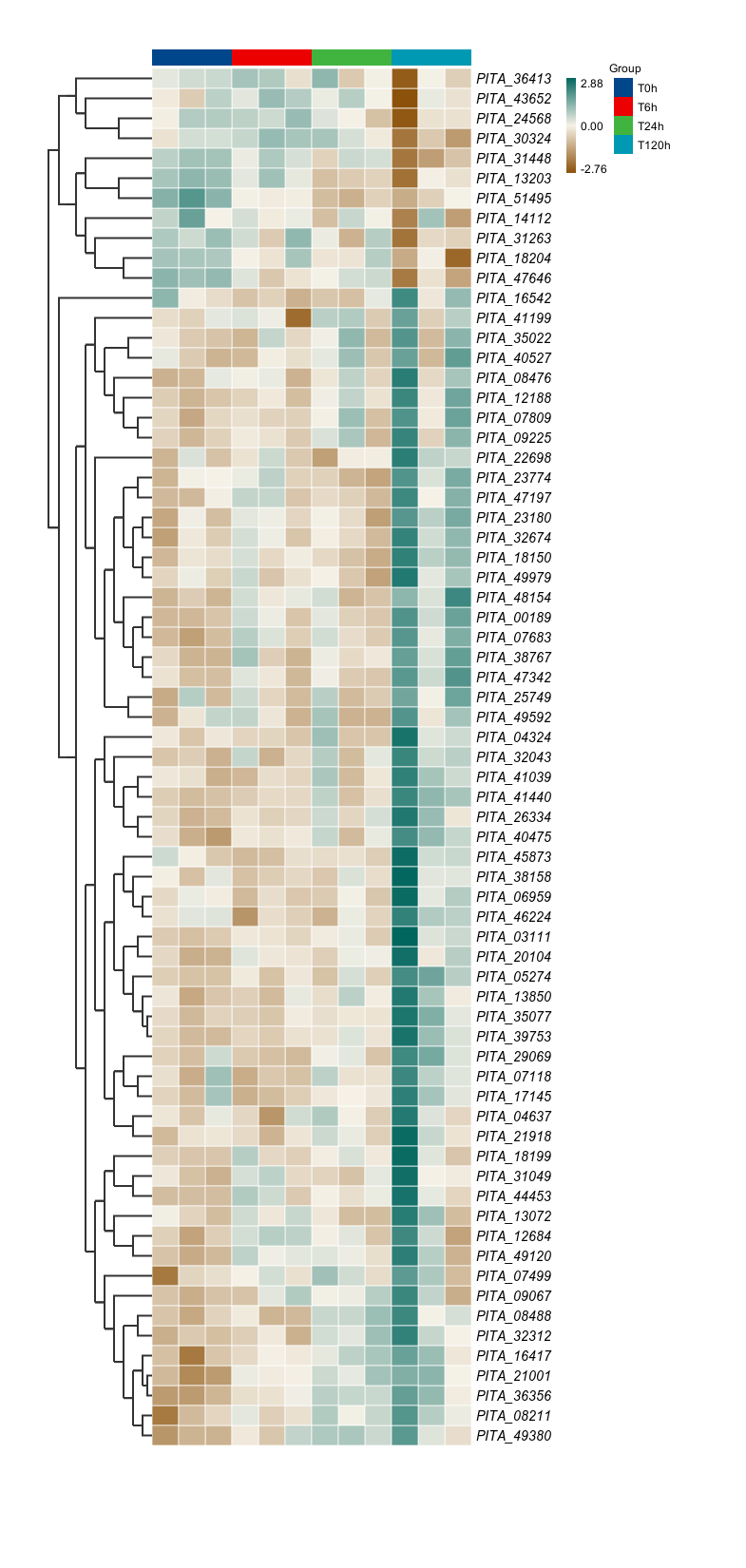

**Supplementary Fig. 2** Heatmap of MEmagenta module core gene expression


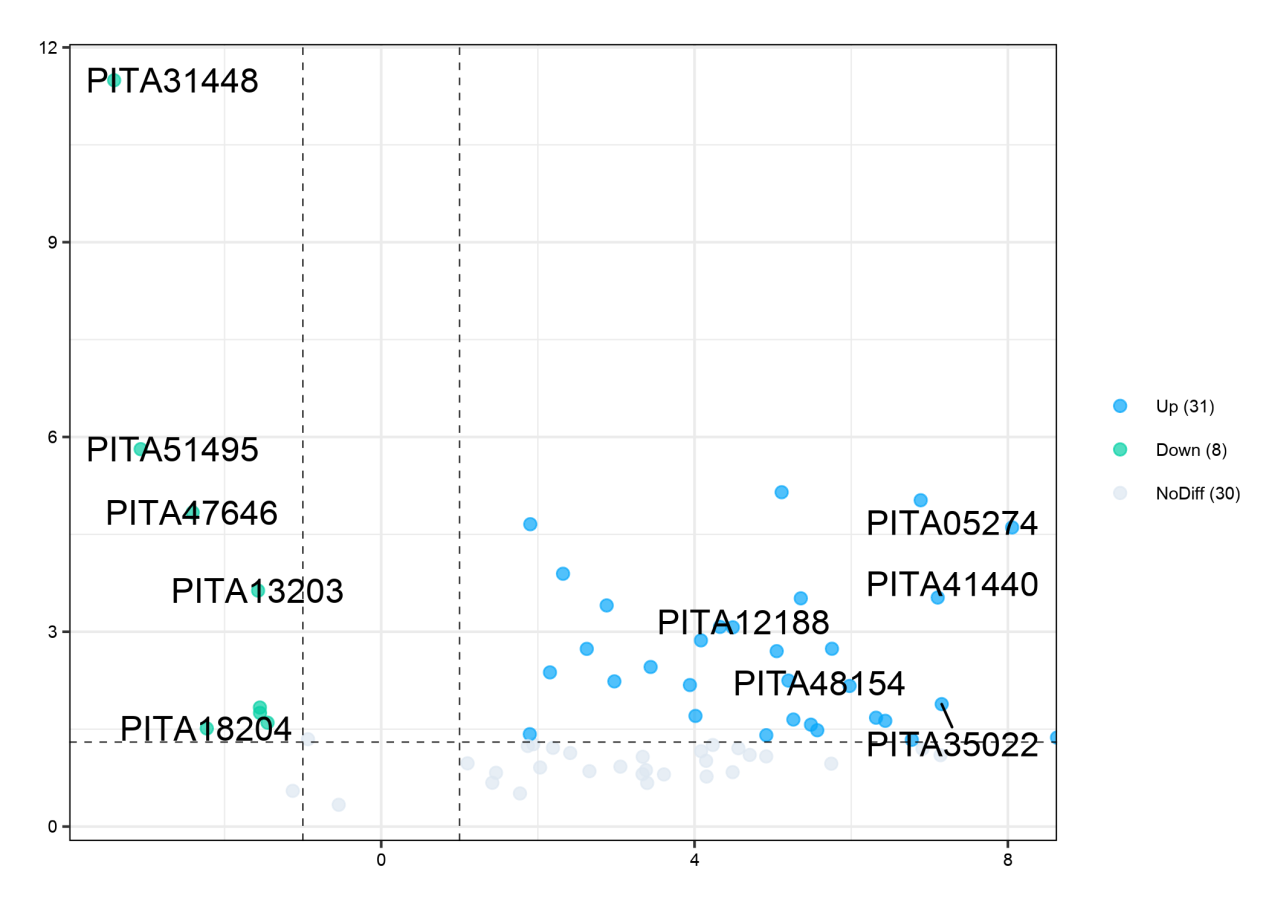


**Supplementary Fig. 3** Selection and analysis of ten genes from the MEmagenta module that exhibit differential expression at random
